# Severe necrotizing sialometaplasia progressing to palatonasal fistula in a patient characterized by habitual self-induced vomiting: a case report

**DOI:** 10.1186/s40337-026-01714-7

**Published:** 2026-07-22

**Authors:** Haruka Yamauchi, Ayaka Matsumoto, Akihiro Dohi, Yuta Inagawa, Shiro Suda

**Affiliations:** 1 Department of Psychiatry, Jichi Medical University, 3311-1 Yakushiji, Shimotsuke-shi, Tochigi, 329-0498 Japan; 2 Department of Dentistry, Oral and Maxillofacial Surgery, Jichi Medical University, Tochigi, Japan

**Keywords:** Eating disorders, Anorexia nervosa, Self-induced vomiting, Necrotizing sialometaplasia, Palatonasal fistula

## Abstract

**Background:**

Self-induced vomiting (SIV) in patients with eating disorders exposes the oral cavity to repeated chemical and mechanical insults, leading to various oral complications. Necrotizing sialometaplasia (NS) is one such complication; however, most cases resolve spontaneously and follow a benign course without requiring surgical intervention.

**Case presentation:**

A woman in her twenties with a long history of an eating disorder characterized by habitual SIV developed NS of the hard palate. Unlike the usual benign course, the lesion failed to heal spontaneously and progressed to a palatonasal fistula. Her clinical course was notable for several behavioral and clinical factors that may have contributed to sustained local irritation of the palatal tissues, such as persistent vomiting, smoking, and dental procedures requiring local anesthesia. Multidisciplinary psychiatric and dental management was provided throughout the clinical course. Since spontaneous closure of the fistula could not be expected, surgical repair was ultimately performed.

**Conclusions:**

Although NS associated with SIV in patients with eating disorders is usually benign and self-limiting, it can rarely progress to severe and irreversible oral complications such as a palatonasal fistula. Despite the fact that NS is not life-threatening, its severe progression can significantly impair quality of life. Accumulating clinical experience with such hidden complications may improve the quality of psychoeducation and advance the treatment of eating disorders.

## Background

Eating disorders accompanied by self-induced vomiting (SIV) are associated with a wide range of medical complications that affect nearly every organ system. Repeated exposure to gastric acid and forceful retching place substantial physiological stress on the body, leading to gastrointestinal injury and characteristic oral and maxillofacial pathologies. In particular, repetitive vomiting results in chronic chemical and mechanical insults to the oral cavity, which may cause dental erosion, mucosal injury, salivary gland disorders, and ulcerative lesions of the palate [1].

Necrotizing sialometaplasia (NS) is a rare, benign inflammatory condition of the minor salivary glands that most commonly affects the hard palate. It has occasionally been reported in patients with eating disorders who engage in habitual SIV [2]. Clinically, NS typically presents as a painful ulcerative lesion and may closely mimic oral malignancies, i.e., squamous cell carcinoma. Although the precise pathophysiology remains incompletely understood, NS is thought to result from ischemic necrosis of the salivary gland tissue. The most frequently reported triggers include local trauma, surgical procedures, and local anesthesia; however, systemic and behavioral factors, such as vomiting, smoking, cocaine use, and radiotherapy, have also been implicated [3–5]. NS is generally self-limiting and typically resolves with supportive care. Surgical intervention beyond biopsy to exclude malignancy is rarely needed; surgical debridement may be considered only in very uncommon cases with extensive lesions [3, 4].

Here, we report a severe case of NS in a patient with an eating disorder characterized by long-standing SIV, in which the lesion progressed to a palatonasal fistula and ultimately required surgical closure. This case highlights the potential for persistent vomiting—particularly in the presence of psychiatric comorbidities—to result in unexpected and irreversible oral complications.

## Case presentation

The patient, an Eastern European woman in her twenties with ADHD, had longstanding school maladjustment and low self-esteem due to ADHD-related difficulties and strict, critical caregiving, and early adverse experiences.At 13 years of age, she developed anorexia nervosa. By the age of 14 years, she had begun habitual SIV. From the age of 20 years, she had smoked approximately 5–15 cigarettes per day.

After relocating to Japan at age 22, she maintained a normal body weight but continued to engage in recurrent binge/purge behaviors consistent with bulimia nervosa. While living in Japan, political instability in her home country exacerbated her depression, and she begun using cannabis to cope with her stress. Three years before presentation, she developed worsening insomnia, for which benzodiazepines were initiated. Afterward, she resumed psychiatric treatment for depression at a local clinic.

**Fig. 1:**
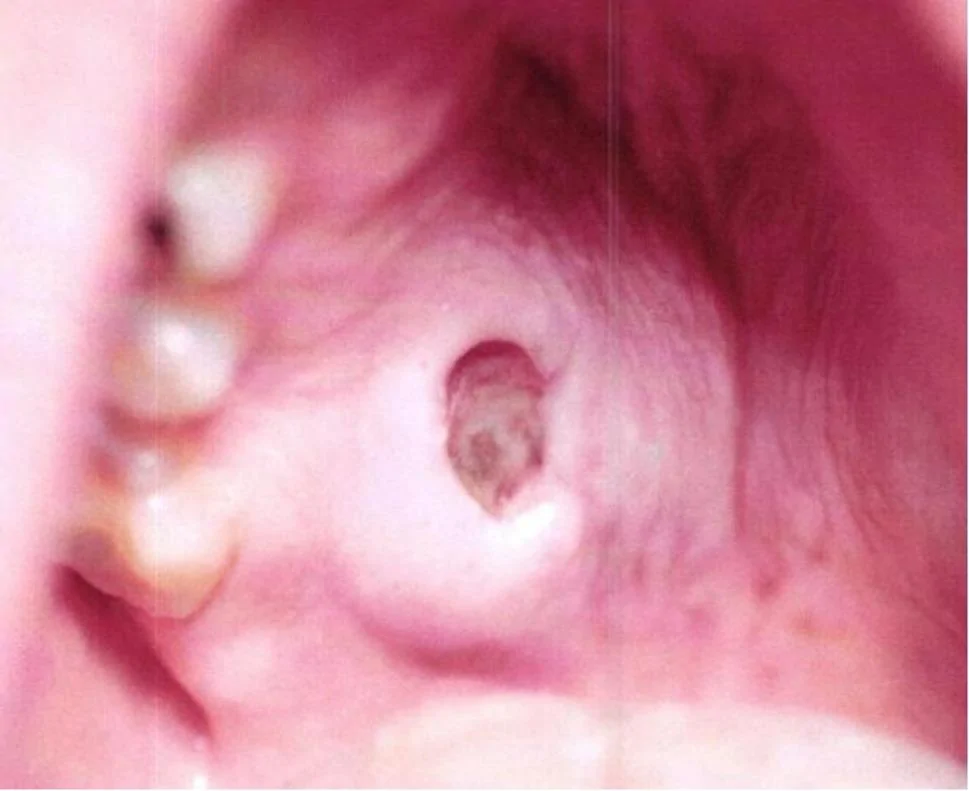
Ulcerative lesion on the right hard palate

**Fig. 2:**
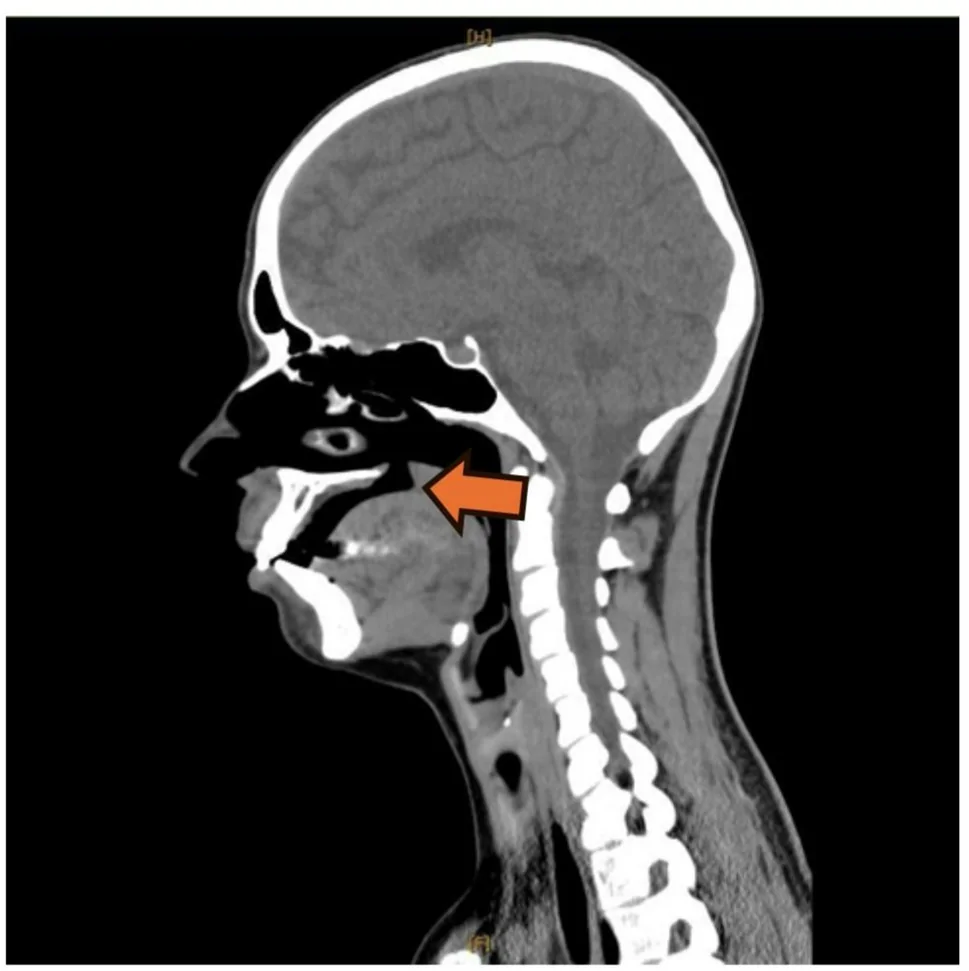
Computed tomography (CT) demonstrating palatonasal fistula formation

**Fig. 3:**
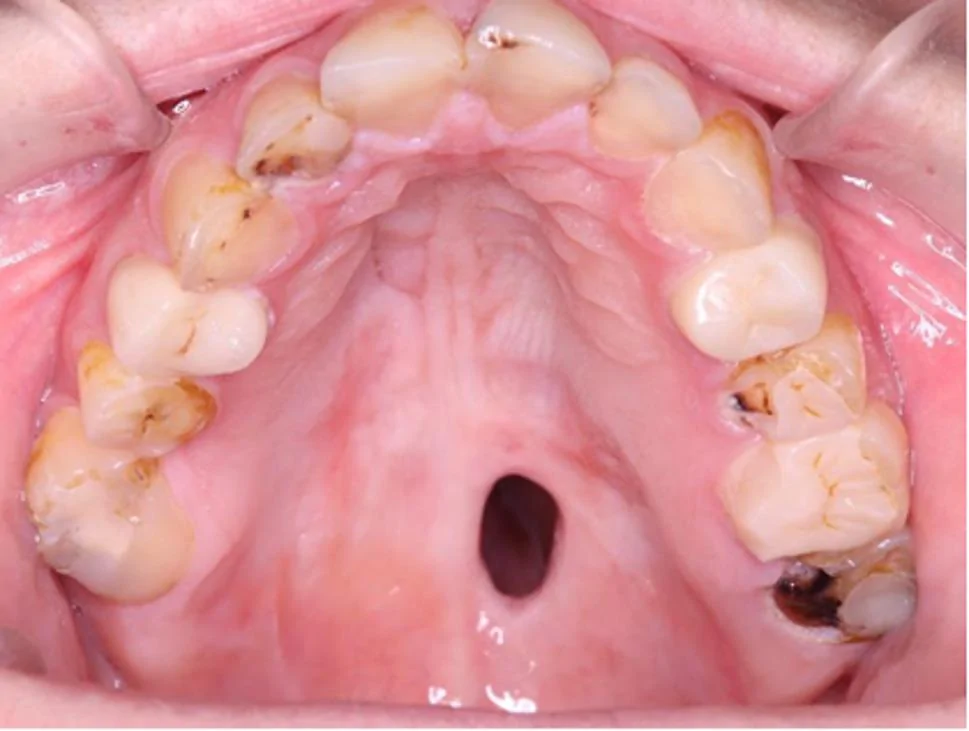
Palatonasal fistula on the left side of the palate

During her course of eating disorder, she needed to visit a dentist due to caries and dental erosion requiring treatment under local anesthesia. She developed pain and swelling in the right hard palate and was referred to an oral surgery department, where biopsy confirmed NS (Fig. 1). The ulcer healed spontaneously. One year before presentation, swelling recurred on the left palate near the prior site and progressed over two weeks to a palatonasal fistula (Figs. 2 and 3). She returned to the oral surgery department with speech difficulties; biopsy again confirmed NS.

Upon presentation at our psychiatric clinic, her height was 168 cm, her weight was 58 kg, and her body mass index (BMI) was 20.6 kg/m². She was taking medications prescribed by her previous physician, including escitalopram (20 mg), brexpiprazole (1 mg), olanzapine (5 mg), trazodone (100 mg), and etizolam (1.5 mg) as regular medications, along with alprazolam (0.8 mg) as needed for anxiety. A chronological summary of the patient’s clinical course is presented in Table 1.

The patient reported an inability to control binge eating and SIV. She reported several binge/purge cycles per day. We considered that psychotropic medications and comorbid ADHD contributed to her difficulty in regulating these behaviors. We suspected that etizolam and alprazolam might have caused disinhibition and impaired her ability to control vomiting impulses, whereas brexpiprazole and olanzapine might have increased her appetite and exacerbated binge-eating impulses. These four medications were gradually tapered and discontinued. Because ADHD-related impulsivity may have amplified her binge–purge impulses, atomoxetine was initiated and titrated to 120 mg. Following the medication adjustments, she was able to control her binge–purge impulses and ceased SIV. At follow-up, her weight had increased to 70 kg, and her BMI was 24.8 kg/m².

**Fig. 4:**
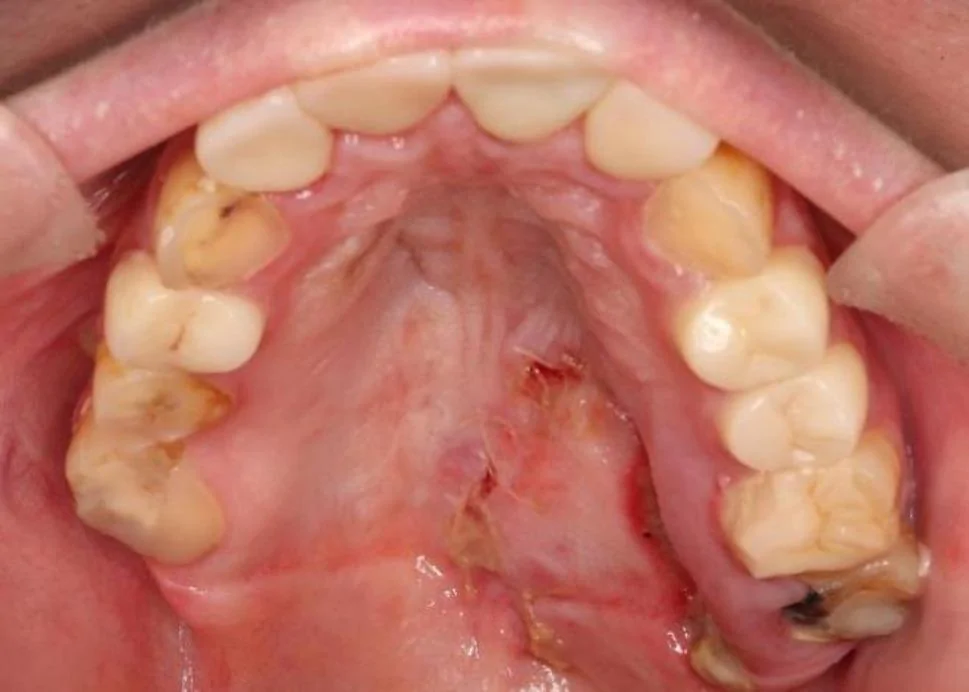
Postoperative closure of the palatonasal fistula

Despite gaining control over her binge–purge behaviors and maintaining cessation of SIV for one year after the initial visit, the palatonasal fistula did not close spontaneously. Given the persistent defect, the oral and maxillofacial surgery team proceeded with surgical closure. Postoperative evaluation one week after the procedure confirmed complete closure of the palatonasal fistula (Fig. 4).

This figure shows the ulcerative lesion that developed on the right posterior hard palate during the initial episode of NS. The lesion appeared after swelling and pain and was diagnosed as NS by biopsy. It subsequently resolved spontaneously without surgical intervention.

The CT image shows a palatonasal fistula extending from the left hard palate to the nasal cavity. The fistulous tract is indicated by an arrow. The lesion developed during the second episode of NS.

This intraoral photograph shows the palatonasal fistula on the left hard palate following recurrent NS. A palatal obturator was placed to improve speech function and reduce nasal regurgitation during eating.

One week after surgical repair, complete closure of the palatonasal fistula is evident. The oral and nasal cavities are fully separated, with well-approximated mucosal tissue and no residual defect.

**Table 1:** Timeline of key clinical events and factors potentially contributing to NS progression

Period	Clinical events	Factors contributing to progression of NS
Age 13	Developed anorexia nervosa, restricting type.	Not applicable.
Age 14	Began habitual SIV; illness transitioned to binge–purge type.	Onset of habitual SIV.
Age 20	Began smoking.	Habitual smoking.
~ 2 years before presentation	Began seeing a local psychiatrist due to worsening depression.	Benzodiazepine and olanzapine use (possible impulse-control impairment).
~ 1 year before presentation	NS developed on the right palate and healed spontaneously.	Ongoing habitual SIV; poor oral hygiene related to depression.
~ 1 year before presentation (later)	Left palatal NS progressed to a palatonasal fistula.	Persistent palatal irritation and impaired local healing capacity.

Chronological summary of the patient’s clinical course, including events and factors potentially associated with the development and progression of NS.

## Discussion and conclusions

This case highlights two important issues. First, NS in a patient with an eating disorder may, although rarely, progress to severe complications such as a palatonasal fistula. Second, psychiatric comorbidities and psychiatric medications may complicate the regulation of SIV and thus contribute to disease severity.

Regarding the first issue, NS arising in patients with eating disorders who engage in habitual SIV can, albeit rarely, progress to a palatonasal fistula. NS is generally a self-limiting condition, and surgical intervention is not considered part of standard management. In rare cases involving unusually extensive necrotic lesions, surgical debridement has been described as an adjunctive measure to facilitate local healing and reduce the risk of secondary infection [3, 4]. Although a case with NS accompanied by bone involvement has been reported, the lesion resolved spontaneously [6]. We found no clearly documented prior case of eating-disorder-associated NS progressing to a persistent palatonasal fistula requiring surgical closure. From an oral and maxillofacial perspective, persistent vomiting, smoking, and dental procedures may have been contributing factors that increased local stress on the palatal tissues, although their precise role in the progression of NS in this case remains uncertain. Although such behaviors are not directly implicated in the pathogenesis of NS, they illustrate how recurrent behaviors, may cause chronic localized injury to the oral mucosa [7, 8].

Regarding the second issue, the patient’s longstanding difficulties with impulse regulation, her history of childhood emotional adversity, and comorbid neurodevelopmental traits constituted a meaningful part of the clinical background. Previous studies have indicated an association between childhood maltreatment and impaired impulse control [9], and psychotropic medications with known effects on appetite or behavioral inhibition—such as second-generation antipsychotics, including olanzapine—have been discussed in relation to binge–purge tendencies in individuals with eating disorders [10]. Disinhibition associated with benzodiazepine use may also intensify binge–purge impulses. Overall, these psychiatric and psychosocial elements provide useful background but do not explain the patient’s persistent binge–purge behaviors or the rare progression of NS to palatonasal fistulation.

Limitations include missing clinical records relevant to defect management and the inability of a single case to determine whether psychiatric comorbidities or medications contributed to persistent vomiting.

In summary, this case demonstrates that NS, although usually self-limiting, can rarely progress to severe complications such as a palatonasal fistula in the context of long-standing self-induced vomiting. While causality cannot be determined, multiple stressors may have contributed. Severe NS can impair quality of life, and accumulating such cases may support better psychoeducation and management of eating disorders.

### Patient perspective

I experienced significant difficulties in daily life after the palatonasal fistula developed, and eventually required surgery to repair it. The discomfort and functional limitations were distressing, and the experience made me determined never to engage in SIV again. After the surgery, I felt relieved knowing that the opening in my palate had been successfully closed.

## Data Availability

No datasets were generated or analysed during the current study.

## Abbreviations

ADHD: Attention-deficit/hyperactivity disorder
BMI: Body mass index
CT: Computed tomography
NS: Necrotizing sialometaplasia
SIV: Self-induced vomiting
